# Calcium Lactate for Lymphatic Headache, Urticaria, Etc.

**Published:** 1909-06-05

**Authors:** 


### CALCIUM LACTATE FOR LYMPHATIC HEADACHE, URTICARIA, Etc.

Urticaria, chilblains, lymphatic headache, and the like are associated with a condition of defective blood coagulability, and it is generally recognised that any lesion that can be included under the general term of serous hæmorrhage can often be greatly benefited by the oral administration of calcium salts. The preparations best adapted for this purpose are the chloride and the lactate, and of these the latter has the advantage that its organic radicle is readily oxidised in the system, with the result that the base remains more fully at the disposal of the organism than is the case with the chloride. It is important, however, that the lactate should be freshly prepared, since it decomposes when kept any length of time. For adults the dose is 15 grains, flavoured with $\frac{1}{2}$ to 1 minim of tincture of capsicum, and made up to the ounce with chloroform water. The treatment should be continued for six weeks at a time, three doses being given daily about an hour before food. More than half the cases of chilblains so treated are rapidly cured, though a repetition of the course may be required the next winter. Other affections in which similar treatment has proved very beneficial are boils, headache of the lymphatic type, urticaria, face-flushings, acne rosacea, and perspiring hands and feet with offensive perspiration. If constipation should result from the use of the calcium, it may be corrected by giving a small dose of infusion of senna pods at bedtime.

